# Comparison of anticoagulation strategies for veno-venous ECMO support in acute respiratory failure

**DOI:** 10.1186/s13054-020-03348-w

**Published:** 2021-01-04

**Authors:** Benjamin Seeliger, Michael Döbler, Robert Friedrich, Klaus Stahl, Christian Kühn, Johann Bauersachs, Folkert Steinhagen, Stefan F. Ehrentraut, Jens-Christian Schewe, Christian Putensen, Tobias Welte, Marius M. Hoeper, Andreas Tiede, Sascha David, Christian Bode

**Affiliations:** 1 Department of Respiratory Medicine, Hannover Medical School and Biomedical Research in End-Stage and Obstructive Lung Disease (BREATH), German Lung Research Center (DZL), Hannover, Germany; 2 Department of Anaesthesiology and Critical Care Medicine, University Hospital Bonn, Bonn, Germany; 3 Department of Gastroenterology, Hepatology and Endocrinology, Hannover Medical School, Hannover, Germany; 4 Department of Cardiothoracic, Transplantation and Vascular Surgery, Hannover Medical School, Hannover, Germany; 5 Department of Cardiology and Angiology, Hannover Medical School, Hannover, Germany; 6 Department of Haematology, Haemostasis, Oncology, and Stem Cell Transplantation, Hannover Medical School, Hannover, Germany; 7 Department of Nephrology and Hypertension, Hannover Medical School, Hannover, Germany; 8 Institute for Intensive Care Medicine, University Hospital Zurich, Zurich, Switzerland

**Keywords:** ECMO, Heparinization, ARDS, Bleeding, Thromboembolism

## Abstract

**Background:**

Extracorporeal membrane oxygenation (ECMO) support in acute respiratory failure may be lifesaving, but bleeding and thromboembolic complications are common. The optimal anticoagulation strategy balancing these factors remains to be determined. This retrospective study compared two institutional anticoagulation management strategies focussing on oxygenator changes and both bleeding and thromboembolic events.

**Methods:**

We conducted a retrospective observational cohort study between 04/2015 and 02/2020 in two ECMO referral centres in Germany in patients receiving veno-venous (VV)-ECMO support for acute respiratory failure for > 24 h. One centre routinely applied low-dose heparinization aiming for a partial thromboplastin time (PTT) of 35–40 s and the other routinely used a high-dose therapeutic heparinization strategy aiming for an activated clotting time (ACT) of 140–180 s. We assessed number of and time to ECMO oxygenator changes, 15-day freedom from oxygenator change, major bleeding events, thromboembolic events, 30-day ICU mortality, activated clotting time and partial thromboplastin time and administration of blood products. Primary outcome was the occurrence of oxygenator changes depending on heparinization strategy; main secondary outcomes were the occurrence of severe bleeding events and occurrence of thromboembolic events. The transfusion strategy was more liberal in the low-dose centre.

**Results:**

Of 375 screened patients receiving VV-ECMO support, 218 were included in the analysis (117 high-dose group; 101 low-dose group). Disease severity measured by SAPS II score was 46 (IQR 36–57) versus 47 (IQR 37–55) and ECMO runtime was 8 (IQR 5–12) versus 11 (IQR 7–17) days (*P* = 0.003). There were 14 oxygenator changes in the high-dose group versus 48 in the low-dose group. Freedom from oxygenator change at 15 days was 73% versus 55% (adjusted HR 3.34 [95% confidence interval 1.2–9.4]; *P* = 0.023). Severe bleeding events occurred in 23 (19.7%) versus 14 (13.9%) patients (*P* = 0.256) and thromboembolic events occurred in 8 (6.8%) versus 19 (19%) patients (*P* = 0.007). Mortality at 30 days was 33.3% versus 30.7% (*P* = 0.11).

**Conclusions:**

In this retrospective study, ECMO management with high-dose heparinization was associated with lower rates of oxygenator changes and thromboembolic events when compared to a low-dose heparinization strategy. Prospective, randomized trials are needed to determine the optimal anticoagulation strategy in patients receiving ECMO support.

## Introduction

In refractory acute respiratory failure (ARF), implementation of veno-venous extracorporeal membrane oxygenation (VV-ECMO) as a rescue strategy may be life-saving and is increasingly applied [1]. However, ECMO support is associated with potentially life-threatening complications, mostly related to either bleeding events or thromboembolic complications [2–4]. To minimize such events, most centres use unfractionated heparin (UFH)-based anticoagulation adjusted by partial thromboplastin time (PTT), usually within 40–80 s or by activated clotting time (ACT) within 140–180 s [5]. Current guidelines advice an ACT-guided approach aiming at 1.5 fold increase of normal [6]. With bleeding complications occurring in up to 50% of patients, there is a quest for alternative anticoagulatory strategies without compromising integrity of the ECMO circuits and risk of thromboembolism [2]. Previous studies found lower heparin dosing to be generally safe with regards to thromboembolic complications but conclusions are limited by small patient numbers [7–9].

Our study aimed to retrospectively compare oxygenator durability, bleeding and thromboembolic events between two experienced ECMO centres with considerably different routine anticoagulation strategies but identical oxygenator change management. We hypothesized, that a strategy including low-dose heparin strategy would result in similar oxygenator durability and similar thromboembolic complications while reducing bleeding events compared to a high-dose strategy.

## Methods

### Design, settings and participants

We conducted a retrospective cohort study including patients with severe ARF receiving VV-ECMO support between April 2015 and February 2020 at two German university hospitals with extensive ECMO experience. Both centres routinely used UFH-based anticoagulation, but with different intensity, thus enabling us to compare a low-dose heparinization strategy aiming for a PTT between 35 and 40 s (measured thrice per day using the actin FS assay by Siemens) with a high-dose heparinization strategy aiming for an ACT between 140 and 180 s (measured every 2 h). In the high-dose-group, PTT was measured once daily using the Pathrombin SL assay, with both tests showing excellent correlation [10]. ECMO systems used were Getinge/Maquet RotaFlow or CardioHelp with cannulation of the internal jugular and/or femoral veins via 19–25 French cannulas. Standard cannulation in the high-dose centre was femoral/jugular venous access, while in the low-dose centre a bi-femoral venous access was mostly established. For the RotaFlow device, the permanent-life-support system was used and for the CardioHelp system the HLS Set Advanced was used. Both systems were manufactured by Getinge, were Bioline-coated and possessed equivalent durability [11]. Patients were identified via established ECMO databases at both sites. Inclusion criteria were VV-ECMO support ≥ 24 h and provided written informed consent by patients or proxy for analysis of clinical data. Exclusion criteria were duration of VV-ECMO support < 24 h; external ECMO support > 24 h before referral; addition of a third (arterial) cannula within 24 h; age < 18 years; acute liver failure with relevant coagulopathy precluding heparin administration; missing informed consent and medical indication for high-dose anticoagulation in the low-dose centre. The study was approved by the institutional review boards at both sites.

### Endpoints

The primary endpoint was ECMO oxygenator change within the first 15 days. Secondary endpoints were 30-day ICU mortality, severe bleeding complications (defined as need for intervention or ≥ 10 red blood cell (RBC) transfusions), symptomatic thromboembolic events, number of platelet and RBC transfusions during ECMO, administered units of UFH during ECMO and coagulation studies (mean ACT in the high-dose-group, mean PTT in both groups).

### Transfusion strategies

Absent of overt bleeding, the routine threshold for RBC transfusion at the high-dose centre was a haemoglobin level < 8 g/dL versus < 9 g/dL in the low-dose group. Routine threshold for platelet transfusions were < 30.000/µL in the high-dose centre versus 70.000/µL in the low-dose centre. With overt bleeding, the routine thresholds for platelet transfusion were 50.000/µL (high-dose centre) versus 100.000/µL (low-dose centre) but could be individualized depending on the clinical scenario, severity and site of bleeding. Antithrombin III was substituted if antithrombin III levels were < 50% and antifibrinolytic agents administered in cases of clinical suspicion of hyperfibrinolysis or proof by thromboelastography. Bleeding management regarding administration of prothrombin complex concentrates was adjusted by event severity at the discretion of the treating physicians and was not standardized in both centres.

### Indication for oxygenator changes

Oxygenator change was considered in the settings of decreasing post-filter pO_2_ < 200 mmHg with increasing transmembrane pressure gradient (with the CardioHelp system), overt circuit thrombosis with thrombi > 5 mm, rising D-dimers with progressive thrombocytopenia and hyperfibrinolysis with increasing transmembrane pressure gradient, and otherwise unexplained haemolysis with increasing transmembrane pressure gradient.

### Covariates

Age, admission and discharge dates to ICU, underlying reason for ARF, body-mass-index, pre-existing antiplatelet therapy and comorbidities were obtained from charts. Simplified acute physiology score (SAPS) II [12] at day of ECMO implantation, Respiratory ECMO Survival Prediction (RESP) score [13], sequential organ failure assessment score (SOFA) [14], heparin doses, ECMO devices and settings and coagulation studies were extracted from the clinical patient data management system.

### Statistical analysis

Continuous data was assessed for normal distribution by Shapiro–Wilk-test and group comparison was performed using t-test or rank-sum test, as appropriate. Hazard ratios for freedom from oxygenator change at 15 days was calculated using a multivariable cox regression. Covariables were selected if baseline values were significantly unbalanced between the groups or if they were overtly physiologically linked to the outcome. We did not include number of blood product transfusions and ECMO device since they were not independent factors but part of the institutional strategy. The final model included age, sex, BMI, RESP Score, SAPS II score, SOFA renal sub-score, ECMO runtime, sepsis, pre-existing coronary artery disease, prior treatment with aspirin, mean ECMO flow and baseline fibrinogen, d-dimers and antithrombin III levels as covariables. Analyses were performed using STATA V16.0 (STATA Corp LP) and RStudio V1.2.5033 (RStudio Inc).

## Results

### Patient characteristics

**Fig. 1:**
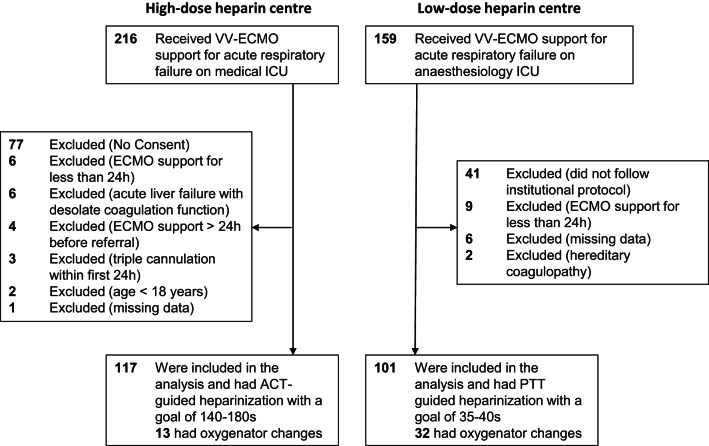
Flow chart for patient cohorts by centre / heparinization strategy. The need for written informed consent at the low-dose centre was waved by the institutional review board due to the retrospective nature of the study

At screening, 375 patients receiving VV-ECMO support for ARF were identified. A total of 218 patients were included in the analysis (117 high-dose group vs. 101 low-dose group), with details on exclusions provided in Fig. 1. The baseline characteristics are shown in Table 1. ECMO settings and associated laboratory are shown in Table 2. The causes of ARF according the RESP score were viral pneumonia (22.5%), bacterial pneumonia (30.3%), status asthmaticus (1.8%), trauma/burn (1.8%), aspiration pneumonia (7.8%), other acute respiratory causes (28.9%) and non-respiratory or chronic respiratory causes (6.8%) and were distributed homogenously among the groups (p = 0.359). Patients in the high-dose group were younger (46 years [IQR 29–55] vs. 54 years [interquartile range (IQR) 44–62], *P* < 0.001) and had a lower BMI (26.2 kg/m^2^ [IQR 22.5–29.4] vs. 29.1 [IQR 26.0–33.2], *P* < 0.001. Sepsis was less frequently present in the high-dose group (75.2 vs. 95.1%, *P* < 0.001), and primary ARDS was more common in the high-dose group (88.9 vs. 68.3%, *P* < 0.001). While the SAPS-II score at day of ECMO implantation was comparable between the groups, the RESP-score was higher in the high-dose group (1 [IQR – 1 to 3] vs. −0 [−2 to 3], *P* = 0.002).

**Table 1:** Baseline characteristics

	ALL (* n* = 218)	High-dose group (* n* = 117)	Low-dose group (* n* = 101)	*P* value
Baseline Characteristics				
Gender, n (%), male	153 (70.2)	85 (72.6)	68 (67.3)	0.392
Age, median (IQR), years	49 (38–60)	46 (29–55)	54 (44–62)	< 0.001
BMI, median (IQR), kg/m^2^	27.7 (24.2–30.9)	26.2 (22.5–29.4)	29.1 (26.0–33.2)	< 0.001
SAPS II (IQR)	47 (37–56)	46 (36–57)	47 (37–55)	0.871
SOFA Score (IQR)	9 (8–10)	9 (7–10)	9 (8–10)	0.650
Respiration-sub-score	4	4	4	
Coagulation-sub-score	0 (0–1)	0 (0–1)	0 (0–2)	0.573
Liver-sub-score	0 (0–1)	0 (0–2)	0 (0–1)	0.059
Cardiovascular-sub-score	4 (3–4)	4 (3–4)	4 (3–4)	0.151
Renal-sub-score	1 (0–4)	1 (0–4)	1 (0–2)	0.004
RESP-Score (IQR)	1 (-2–3))	1 (-1–3)	0 (-2–3)	0.002
Sepsis, n (%)	184 (84.4)	88 (75.2)	96 (95.1)	< 0.001
Primary ARDS, n (%)	173 (79.4)	104 (88.9)	69 (68.3)	< 0.001
Immunocompromised, n (%)	68 (31.2)	31 (26.5)	37 (36.6)	0.107
Liver failure*	53 (24.3)	34 (29.1)	19 (18.8)	0.079
*Comorbidities, n (%)*				
Diabetes mellitus	42 (19.4)	19 (16.4)	23 (22.8)	0.234
Arterial hypertension	76 (35.0)	34 (29.3)	42 (41.6)	0.059
Coronary artery disease	21 (9.6)	7 (6.0)	14 (13.9)	0.049
Malignancy (solid)	8 (3.7)	2 (1.7)	6 (5.9)	0.098
Malignancy (Hematologic)	14 (6.4)	8 (6.8)	6 (5.9)	0.788

*ARDS* acute respiratory distress syndrome, *BMI* Body mass index, *ECMO* extracorporeal membrane oxygenation, *IQR* interquartile range, *RESP* Respiratory ECMO Survival Prediction score, *SAPS II* Simplified acute physiology score II, *SOFA *sequential organ failure assessment

^*^Defined by sequential organ failure assessment liver subscore of ≥ 2

**Table 2:** ECMO settings and relevant coagulation factors

	ALL (* n* = 218)	High-dose group (* n* = 117)	Low-dose group (* n* = 101)	*P* value
ECMO device				< *0.001*
CardioHelp		31 (26.5)	99 (98)	
RotaFlow		86 (73.5)	2 (2)	
Canula site out				
Jugular vein	3 (1.4)	3 (2.6)	0	
Femoral vein	215 (98.6)	114 (97.4)	101 (100)	
Canula site in				
Jugular vein	135 (61.9)	113 (96.6)	22 (21.8)	
Subclavian vein	2 (0.9)	2 (1.7)	0	
Femoral vein	81 (37.2)	2 (1.7)	79 (78.2)	
Canula size out	23 (23–35)	23 (23–23)	25 (25–25)	< *0.001*
Canula size in	17 (17–23)	17 (17–17)	23 (21–25)	< *0.001*
ECMO runtime, median (IQR), days	9 (5–14)	8 (5–12)	11 (7–17)	0.003
ECMO flow, median (IQR), liter per minute	3.8 (3.3–4.4)	3.5 (2.8–3.9)	4.4 (3.8–4.9)	< 0.001
Days from mechanical ventilation to ECMO implantation, median (ICR)	1 (0–3)	1 (0–3)	1 (0–4)	0.185
Antiplatelet therapy pre-ECMO				
Aspirin	31 (14)	13 (11)	18 (18)	*0.157*
P2Y_12_-inhibitors	6 (3)	2 (2)	4 (4)	*0.311*
Antiplatelet therapy during ECMO, n (%)				
Aspirin	27 (12)	9 (8)	18 (18)	*0.024*
P2Y_12_-inhibitors	5 (2)	0	5 (5)	*0.015*
Baseline fibrinogen, g/L (IQR)	4.7 (3.3–6.2)	6.7 (3.3–6.4)	4.8 (3.4–6.1)	*0.611*
Minimal fibrinogen, g/L (IQR)	2.2 (1.4–3.4)	2.5 (1.6–3.5)	2.0 (1.2–3.4)	*0.030*
Baseline d-dimers, mg/L (IQR)	7.6 (3.6–15.4)	5.4 (2.6–12.7)	8.3 (4.4–16.5)	*0.010*
Maximum d-dimers, mg/L (IQR)	30 (19.3–35)	28.5 (13–30)	35 (33.4–35)	< *0.001*
Baseline antithrombin III, % (IQR)	67 (53–86)	79 (60–93)	60 (44–75)	< *0.001*
Minimum antithrombin III, % (IQR)	59 (45–76)	69 (56–84)	48 (37–62)	< *0.001*
Antithrombin III substitution, n (%)	29 (13.3)	8 (6.8)	21 (21)	*0.002*
Baseline thrombocyte count, thousand / µL (IQR)	174 (101–265)	167 (109–269)	183 (97–263)	*0.878*
Minimum thrombocyte count, thousand / µL (IQR)	62 (36–88)	65 (33–106)	60 (40–81)	*0.868*
Received PCC, n (%)	36 (16.5)	8 (6.8)	26 (25.7)	< *0.001*
Received tranexamic acid, n (%)	113 (51.8)	61 (52.1)	52 (51.5)	*0.924*

*ECMO* extracorporeal membrane oxygenation, *IQR* interquartile range, *PCC* prothrombin complex centrates

#### Primary endpoint

**Fig. 2:**
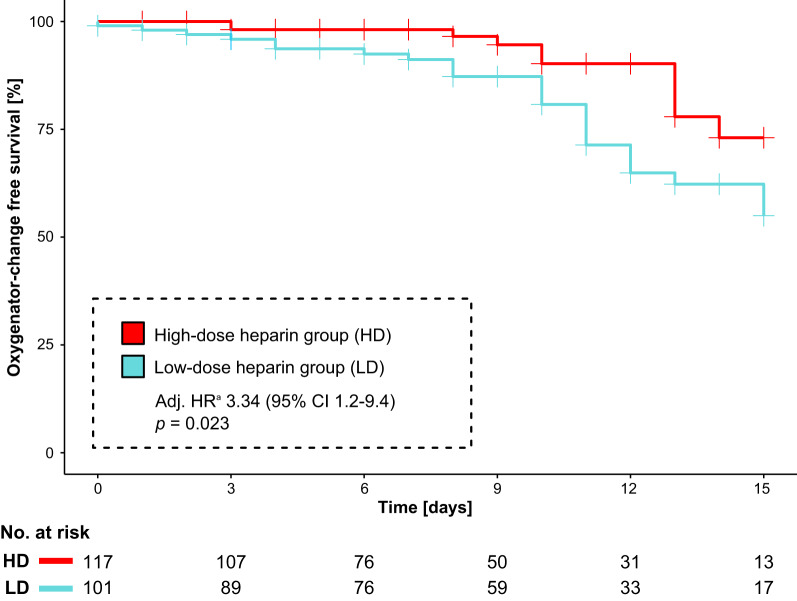
Freedom of oxygenator-change at 15 days between high-dose and low-dose heparinization groups. Data is censored for decannulation or mortality. *HR* hazard ratio, *CI* confidence interval. ^a^Adjusted for age, gender, body-mass-index, Respiratory ECMO Survival Prediction (RESP) score, Simplified acute physiology score II, sequential organ failure assessment renal sub-score, ECMO runtime, sepsis, coronary artery disease, prior aspirin use, mean ECMO blood flow, baseline fibrinogen, d-dimer and antithrombin III levels.

**Fig. 3:**
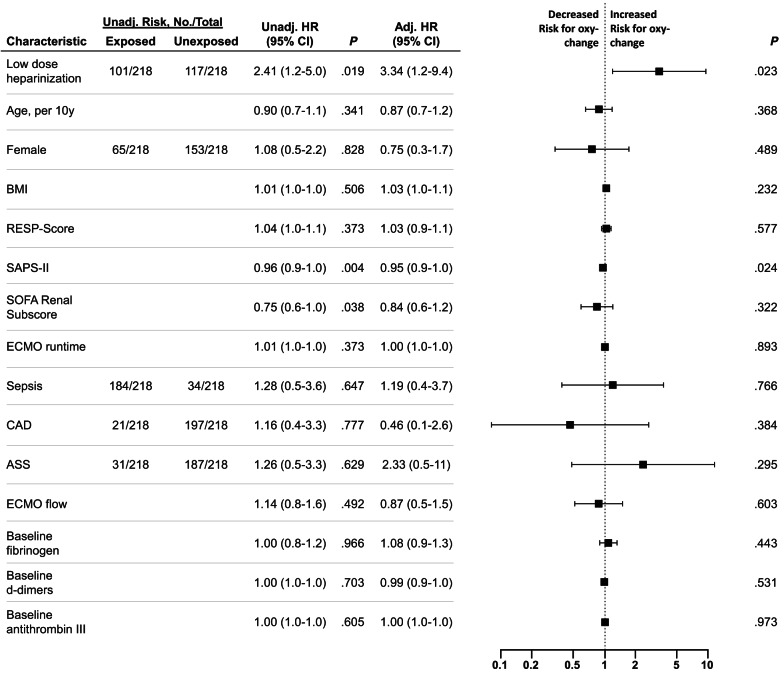
Multivariable cox regression model for oxygenator change. *BMI* body mass index, *CAD* coronary artery disease, *RESP* Respiratory ECMO Survival Prediction score, *SAPS* simplified acute physiology score, *SOFA* sequential organ failure assessment

Overall, there were 14 oxygenator changes in the high-dose group versus 48 in the low-dose group in 13 versus 32 patients. Freedom from oxygenator change at 15 days was 73% in the high-dose group versus 55% in the low-dose group (adjusted hazard ratio (HR) 3.34 [95% confidence interval 1.2–9.4 with low-dose heparinization], *P* = 0.023) (Fig. 2), with the entire regression model displayed in Fig. 3. Reasons for oxygenator changes were decreasing post-filter paO_2_ (86% and 59%), thrombus formation with increasing D-dimers (14% and 39%), and overt haemolysis (0% and 2%). The results were similar when analysing freedom from oxygenator change without censoring data at 15 days (adjusted HR 3.28 [95% confidence interval 1.2–8.6] with low-dose heparinization, *P* = 0.016).

#### Secondary endpoints

**Fig. 4:**
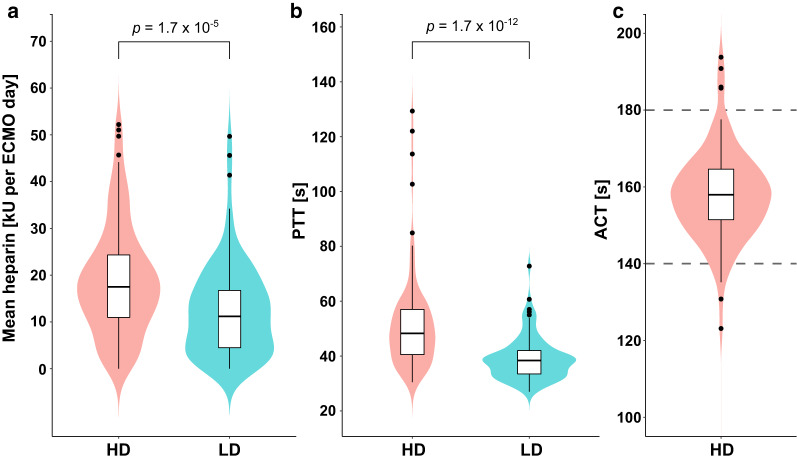
Mean units of UFH administered per day (**a**); mean partial thromboplastin time (PTT) during ECMO (**b**) and mean activated clotting time (ACT) in the high-dose (HD) UFH group (**c**). *ECMO* extracorporeal membrane oxygenation, *HD* high dose heparin groups, *LD* low dose heparin group, *PTT* partial thromboplastin time, *ACT* activated clotting time, *UFH* unfractionated heparin

The overall median of the individual mean ACT in the high-dose group was 158 s (IQR 151–165) and the median of the mean PTT in the high-dose group was 48 s (IQR 41–57) versus 38 s (IQR 34–42) in the low-dose group (*P* < 0.001) (Fig. 4b, c). The corresponding mean units of heparin administered while on ECMO were 17,495 (IQR 10,971–24,327) vs. 11,185 (IQR 4,372–16,750) (*P* < 0.001) (Fig. 4a). The centre-defined ACT and PTT corridors were well-represented in the groups. Additional baseline coagulation parameters are shown in Table 2. In the patients who received an oxygenator change, the d-dimer foldchange compared to baseline was 2.97 (IQR 1.5–8.2) compared to 1.5 (IQR 0.4–4.5), in patients who did not undergo oxygenator change (*P* = 0.002).

Severe bleeding was not different and occurred in 23 (19.7%) of patients in the high-dose group and in 14 (13.9%) in the low-dose group (*P* = 0.256) (Table 3). Of note, severe intracranial bleeding only occurred in the high-dose group with fatal outcome in 5 of 7 cases, while 3 events of intracranial bleeding in the low-dose group were incidental findings on CT without overt neurological deficit (and where thus not categorized as severe bleeding events).

**Table 3:** Secondary outcomes

	ALL (*n* = 218)	High-dose group (*n* = 117)	Low-dose group (*n* = 101)	*P* value
*Severe bleeding events, n (%)*	37 (17.0)	23 (19.7)	14 (13.9)	0.256
Gastrointestinal / intrabdominal	12 (5.5)	5 (4.3)	7 (6.9)	
Intracranial	7 (3.2)	7 (6.0)	0	
Intrathoracic	16 (7.3)	10 (8.5)	6 (5.9)	
Cannula site	2 (0.9)	1 (0.9)	1 (1.0)	
Ear-nose-throat	1 (0.5)	1 (0.8)	0	
RBC transfusions	7 (3–13)	6 (2–10)	8 (6–19)	< 0.001
Platelet transfusions	1 (0–5)	0 (0–1)	4 (0–10)	< 0.001
*Thromboembolic events, n (%)*	27 (12.4)	8 (6.8)	19 (19)	0.007
Intraabdominal embolism / thrombosis	5 (2.3)	3 (2.5)	2 (2.0)	
Venous thrombosis	12 (5.5)	4 (3.4)	7 (6.9)	
Pulmonary embolism	3 (1.8)	1 (0.9)	2 (2.0)	
Peripheral arterial embolism	2 (0.9)	0	2 (2.0)	
ECMO filter embolism	3 (1.8)	0	3 (3.0)	
ECMO cannula thrombosis	1 (0.5)	0	1 (1.0)	
Ischemic stroke	2 (0.9)	0	2 (2.0)	
Heparin-induced thrombocytopenia, n (%)	3 (1.4)	1 (0.9)	2 (2.0)	
30-day ICU mortality	70 (32.1)	39 (33.3)	31 (30.7)	0.110
Overall ICU mortality	91 (41.7)	47 (40.2)	44 (43.6)	0.612

*ECMO* extracorporeal membrane oxygenation, *ICU* intensive care unit, *RBC* red blood cells, *UFH* unfractionated heparin

Applying different in-house standards of transfusion procedures, the number of RBC unit transfusions were significantly lower in the high-dose group compared to the low-dose group (6 [IQR 2–10] vs. 8 [IQR 6–19] *P* < 0.001), as were units of platelet transfusions (0 [IQR 0–1] vs. 4 [0–10], *P* < 0.001). More patients in the low-dose group received prothrombin complex concentrates (26 [25.7%] vs. 8 [6.8%], p < 0.001) and antithrombin III substitution (21 [21%] vs. 8 [6.9%], p = 0.002), while administration of tranexamic acid was similar (Table 2). All patients who received prothrombin complex concentrates had severe coagulopathy in the context of planned intervention with high bleeding risk (high-dose group: 2/8; low-dose group: 15/26) or significant haemoglobin-relevant bleeding (high-dose group: 6/8; low-dose group 11/26). Pre-ECMO use of antiplatelet therapy was not associated with oxygenator change or severe ECMO-related bleeding events.

Fewer thromboembolic events occurred in the high-dose group (8 [6.8%]) than in the low-dose group (19 [19%], *P* = 0.007). Of note, direct thrombotic events of the ECMO circuit (cannula thrombosis *n* = 1; coagulation of the oxygenator *n* = 2) occurred only in the low-dose group (Table 3).

The 30-day ICU mortality was comparable with 33.3% (*n* = 39) in the high-dose versus 30.7% (*n* = 31) in the low-dose group (*P* = 0.11). The main reasons for mortality in the high-dose versus low-dose group were cessation of therapy due to medical futility in 23 (59%) versus 12 (38.7%); refractory multiorgan failure in 11 (28.2%) versus 18 (58.1%), intracranial bleeding in 5 (12.8%) versus 0; abdominal bleeding complications in 0 versus 1 (3.2%).

### Discussion

The key finding of this study is that when compared to an ACT-guided high dose heparinization strategy aiming for 140–180 s, a low dose heparin strategy adjusted by PTT aiming for 35–40 s is associated with a three-fold higher need for oxygenator changes during VV-ECMO support. Although all oxygenator changes in this study were uneventful, these procedures are resource-intensive and may be potentially life-threatening in patients fully dependent on ECMO support.

We initially hypothesized that lower heparin doses might be as efficient as therapeutic high dose anticoagulation regarding ECMO oxygenator durability with similar rates of bleeding events and thromboembolic events. However, in our study, low-dose anticoagulation was not only associated with a higher need for oxygenator changes but also with a higher rate of thromboembolic events. Contrarily, bleeding complications, foremost intracerebral bleeding events were less common in the low-dose group.

Between the two centres, there were overt and significant differences regarding number of transfusions for both RBC and platelet transfusions with considerably greater amounts given in the low-dose group. It is important to point out that these changes rather reflect the more liberal transfusion strategy in the low-dose centre than bleeding severity. At the same time, prothrombin complex concentrates and antithrombin III preparations were also more commonly administered in the low-dose group, where peri-operative patients were treated more often. In studies unrelated to ECMO, RBC transfusion have been shown to increase platelet responsiveness especially with decreased platelet counts and overall incidence of thromboembolic events [15–17]. With ECMO support, both transfusions of platelets and RBC have been reported as independent risk factors for mortality [18, 19]. Therefore, differences in the transfusion strategy might have influenced the outcome in the current study and are not exclusively explained by heparinization. Future prospective RCT evaluating the optimal anticoagulation strategy in patients receiving ECMO support should be planned with pre-specified transfusions strategies.

The findings from our study contradict previous results from a small prospective trial (*n* = 10) where heparinization aiming for a PTT of 45–55 s (vs. 35–40 s in the present study) was compared to a standardized dose of 10U / kg / hour summing up to comparable mean doses in the high-dose group of the present study [20] showing no differences in oxygenator changes and bleeding events with considerable chances for underpowering. A longitudinal single-centre pre-post designed retrospective trial (*n* = 40) showed similar survival to decannulation rates, bleeding events and thromboembolic complications [21], but interestingly the ACTs in both groups were rather high (167 s vs. 189 s) compared to our cohort. Another mixed cohort including 22 patients with VV-ECMO compared heparinization guided by ACT (140–160 s vs. 180–220) and consistently found fewer bleeding events and similar rates of oxygenator changes [22], also aiming for higher ACT-prolongation than in our cohort. The lack of uniform anticoagulation strategies and outcome definitions across all studies render comparison of event rates difficult [23].

Beside mere heparin dosage, temporary interruption of heparin (e.g. in response to bleeding) may create a hypercoagulable milieu, thereby increasing the risk of clotting and oxygenator failure [24, 25], which might have influenced the results of the present study. Case series and smaller retrospective studies reported feasibility of heparin-free ECMO support in cases of trauma or severe bleeding [26–29] and intermittent subcutaneous administration of heparin to avoid heparin pauses [25], but the optimal management strategies in these particularly challenging situations needs to be prospectively investigated and lies beyond the scope of the current retrospective study.

In recent years, more centres integrated anti-Xa levels alongside ACT or PTT in their routine coagulation monitoring during ECMO support following promising results mainly in paediatric populations and better reflection of heparin concentrations [30–33]. Since inflammation can influence ACT measurements [34] the ECMO centres of the current study also utilize anti-Xa levels alongside thromboelastography and single clotting factor analysis where coagulation state is uncertain, and PTT or ACT seems out of line with heparin dosing. However, longer turn-around time and varying 24/7 availability of anti-Xa measurements are limiting factors and 97% of ECMO centres were still using ACT for heparin dose adjustments in 2014 [35]. Since current ELSO guidelines recommend ACT and PTT for measuring heparin effects [6], the analysis of different heparinization strategies based on these assays provide valuable information for intensivists caring for ECMO patients. Yet, we acknowledge that anti-Xa levels may be a more appropriate measurement of heparin effects than ACT or PTT in critically ill patients.

Limitations of this study were inherent to the retrospective design and the comparison of two centres, which render the data subject to substantial potential bias, including different bleeding management strategies, different ECMO devices, cannulation sites and patient populations. Our data thus needs prospective validation with uniform strategies for bleeding management, transfusion strategies and ECMO configuration to derive solid recommendations.

### Conclusion

In this two-centre cohort study, the institutional strategy with a high-dose heparinization during ECMO support was associated with lower rates of oxygenator changes and thromboembolic events, compared to the strategy with low-dose heparinization. Prospective randomized validation with standardized bleeding management and ECMO settings is needed to confirm these findings.

## Data Availability

The data used for this research are available from the corresponding author on reasonable request and subject to Institutional Review Board guidelines.

## Abbreviations

ACT: Activated clotting time
ARDS: Acute respiratory distress syndrome
ARF: Acute respiratory failure
ECMO: Extracorporeal membrane oxygenation
HR: Hazard ratio
ICU: Intensive care unit
IQR: Interquartile range
PTT: Partial thromboplastin time
RBC: Red blood cells
RESP: Respiratory ECMO Survival Prediction
SAPS: Simplified acute physiology score
SOFA: Sequential organ failure assessment
UFH: Unfractionated heparin
VV-ECMO: Venovenous extracorporeal membrane oxygenation
